# Exploring the Anti-Cancer Effects of Fish Bone Fermented Using *Monascus purpureus*: Induction of Apoptosis and Autophagy in Human Colorectal Cancer Cells

**DOI:** 10.3390/molecules28155679

**Published:** 2023-07-27

**Authors:** Ya-Ting Chen, Shu-Jen Chen, Chun-Yi Hu, Cheng-Di Dong, Chiu-Wen Chen, Reeta Rani Singhania, Shu-Ling Hsieh

**Affiliations:** 1 Department of Seafood Science, National Kaohsiung University of Science and Technology, Kaohsiung 81157, Taiwan; melodyyu.chen@gmail.com; 2 Department of Chemical and Materials Engineering, National Kaohsiung University of Science and Technology, Kaohsiung 80778, Taiwan; biochen@nkust.edu.tw; 3 Department of Food Science and Nutrition, Meiho University, Pingtung 912009, Taiwan; cyhu03@ntu.edu.tw; 4 Department of Marine Environmental Engineering, National Kaohsiung University of Science and Technology, Kaohsiung 81157, Taiwan; cddong@nkust.edu.tw (C.-D.D.); cwchen@nkust.edu.tw (C.-W.C.); reetasinghania@nkust.edu.tw (R.R.S.)

**Keywords:** fish bone, *Monascus purpureus*, apoptosis, autophagy, anti-cancer

## Abstract

Fish bone fermented using *Monascus purpureus* (FBF) has total phenols and functional amino acids that contribute to its anti-oxidant and anti-inflammatory properties. Colorectal cancer, one of the most prevalent cancers and the third largest cause of death worldwide, has become a serious threat to global health. This study investigates the anti-cancer effects of FBF (1, 2.5 or 5 mg/mL) on the cell growth and molecular mechanism of HCT-116 cells. The HCT-116 cell treatment with 2.5 or 5 mg/mL of FBF for 24 h significantly decreased cell viability (*p* < 0.05). The S and G2/M phases significantly increased by 88–105% and 25–43%, respectively (*p* < 0.05). Additionally, FBF increased the mRNA expression of caspase 8 (38–77%), protein expression of caspase 3 (34–94%), poly (ADP-ribose) polymerase (PARP) (31–34%) and induced apoptosis (236–773%) of HCT-116 cells (*p* < 0.05). FBF also increased microtubule-associated protein 1B light chain 3 (LC3) (38–48%) and phosphoinositide 3 kinase class III (PI3K III) (32–53%) protein expression, thereby inducing autophagy (26–52%) of HCT-116 cells (*p* < 0.05). These results showed that FBF could inhibit HCT-116 cell growth by inducing S and G2/M phase arrest of the cell cycle, apoptosis and autophagy. Thus, FBF has the potential to treat colorectal cancer.

## 1. Introduction

Colorectal cancer is one of the highest-risk human malignancies. The incidence of colorectal cancer worldwide is increasing, which is attributable to lifestyles, such as obesity, physical inactivity, an unbalanced diet and smoking [1]. In addition, after colorectal cancer, therapy often appeared following a poor prognosis and high recurrence and metastasis rates [2]. Therefore, effective and safe strategies to reduce the incidence of colorectal cancer are required.

Effective cancer treatment methods include encouraging apoptosis and autophagy in cancer cells, as well as inducing cell cycle arrest [3,4]. Cell proliferation and cell growth are coordinated processes that are controlled by the G0, G1, S, G2 and M stages of the cell cycle [5]. However, the most often noticed characteristic in the growth of cancer is avoiding cell cycle arrest. Therefore, inhibition of cancer cell growth can be achieved through cell cycle arrest [6,7,8]. Apoptosis can be induced by either surface death receptors or the mitochondrial pathway, which then cause the Bcl-2 family to be regulated and the caspase family to be activated, resulting in the death of the cell [9]. In autophagy, it can be triggered either by PI3K/Akt/mTOR or by regulating the beclin 1 and phosphoinositide 3 kinase class III (PI3K III), thereby activating microtubule-associated protein 1B light chain 3 (LC3) to form autolysosomes fused with lysosomes, which eventually leads to cellular autodegradation [10,11].

Dietary substances can enhance anticarcinogenic activity and modulate physiological functioning, according to medical and nutritional science [12]. Fermentation has long been utilized for food production and human consumption. It is not only beneficial for prolonging the shelf life of foods but can also increase their nutritional value, including amino acids, essential fatty acids and vitamin contents in a safe and effective manner [13]. Furthermore, fermented foods can possess biological activities that include anti-aging, anti-oxidant, anti-cancer and anti-obesity properties [13]. Studies have shown that rice [14], coix seed [15] and sorghum liquor biowaste [16] fermented with *Monascus* have anti-cancer activity. Our earlier research demonstrated that fish bone fermented with *Monascus purpureus* (*M. purpureus*) (FBF) can enhance anti-oxidant ability and reduce inflammatory effects, which is attributable to increased total phenols and functional amino acid content by fermentation [17,18]. However, the anti-cancer effects of FBF and their mechanisms of action have not been studied.

Our previous studies have shown that FBF obtained from fish bones fermented with *Monascus purpureus* increases the content of soluble protein, peptides and total phenolics and significantly increases the content of total free amino acids. In addition, it has also been proven to have antioxidant and anti-inflammatory properties [17,18]. In the present study, we investigated the cellular anti-cancer mechanisms of *Monascus purpureus* (FBF)- fermented fish bone underlying the induction of cell cycle arrest, apoptosis and autophagy in human colorectal cancer cells (HCT-116 cells).

## 2. Results

### 2.1. Effects of FBF on Cell Viability

**Figure 1:**
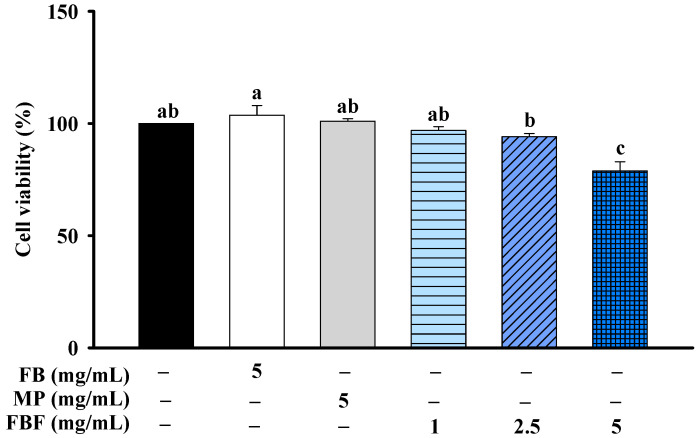
Effect of FB, MP or FBF on cell viability in HCT-116 cells. Data are expressed as mean ± SD (*n* = 5). Different letter means that the values significantly differ from the others at *p* < 0.05 (Tukey’s test). FB: Fish bone; MP: *Monascus purpureus*; FBF: fish bone fermented with *Monascus purpureus* for 3 days.

MTT assays were used to evaluate the cell viability of fish bone (FB), *Monascus purpureus* (MP), and FBF on HCT-116 cells. The cells were treated with FB (5 mg/mL), MP (5 mg/mL) or FBF (1 mg/mL) for 24 h; they were not significantly different from that of control cells. However, after being treated with 2.5 or 5 mg/mL of FBF for 24 h, the cell viability was considerably lower than those of control cells (Figure 1).

**Figure 2:**
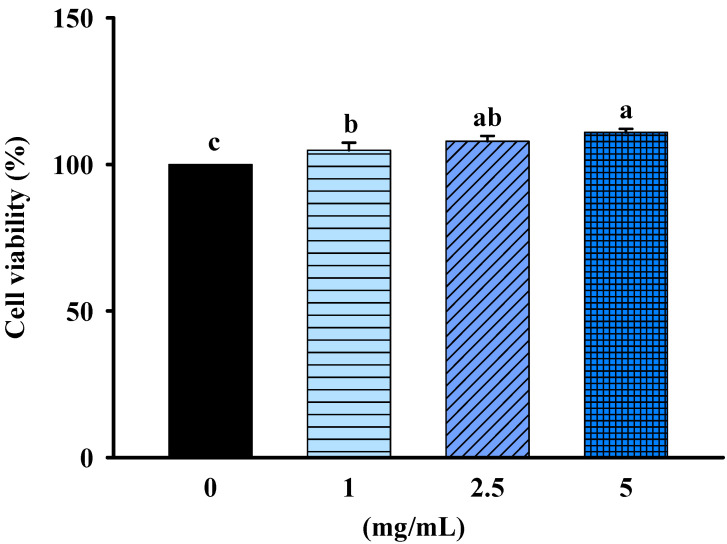
Effect of FBF on cell viability in NRK-52E cells. Data are expressed as mean ± SD (*n* = 5). Different letter means that the values significantly differ from the others at *p* < 0.05 (Duncan’s multiple range tests). FBF: fish bone fermented with *Monascus purpureus* for 3 days.

Safety analysis and toxicity profiling of FBF on non-cancerous cells (rat kidney epithelial cell, NRK-52E cells) was determined by MTT assays. The cells were treated with 1, 2.5 or 5 mg/mL of FBF for 24 h; they were not significantly different from that of control cells. The results indicated that FBF was safe and had no cytotoxicity in NRK-52E cells (Figure 2).

### 2.2. FBF Inhibits the Growth of HCT-116 Cells by Inducing S and G2/M-Phase Arrest

**Figure 3:**
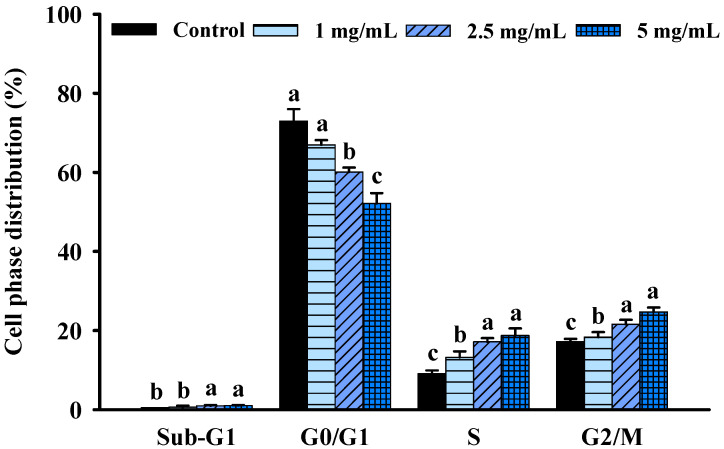
Effect of FBF on cell distribution percentage in each phase of the cell cycle in HCT-116 cells. Data are expressed as the mean ± SD (*n* = 5). Different letter means that the values significantly differ from the others at *p* < 0.05 (Duncan’s multiple range tests). FBF: fish bone fermented with *Monascus purpureus* for 3 days.

Cell cycle progression is one of the most essential needs for growth and proliferation. However, growth inhibition is frequently linked to cell cycle arrest. To further understand the mechanisms of FBF-mediated reduction in HCT-116 cell proliferation, the cell cycle changes following FBF therapy were examined using a NucleoCounter^®^ NC-3000^TM^ fluorescence imaging cytometer. As shown in Figure 3 and Figure S1, different cell-cycle phases responded differently to FBF treatments, with a reduction in the fraction of cells in the G0/G1 phase. However, the S and G2/M phases significantly increased after FBF treatments. The percentage of cells in the G0/G1 phase was 67.0, 60.1 and 52.2% of FBF-treated HCT-116 cells for doses of 1, 2.5 or 5 mg/mL, respectively; it was 74.0% in the control group. Histograms showed a significantly increased percentage of cells in the S phase (13.2~18.7%) and the G2/M phase (18.3~24.7%) with a different concentration FBF treatment when compared to that of the control cells in the S phase (9.1%) and the G2/M phase (17.2%).

### 2.3. FBF Inhibits the Growth of HCT-116 Cells by Inducing Apoptosis

**Figure 4:**
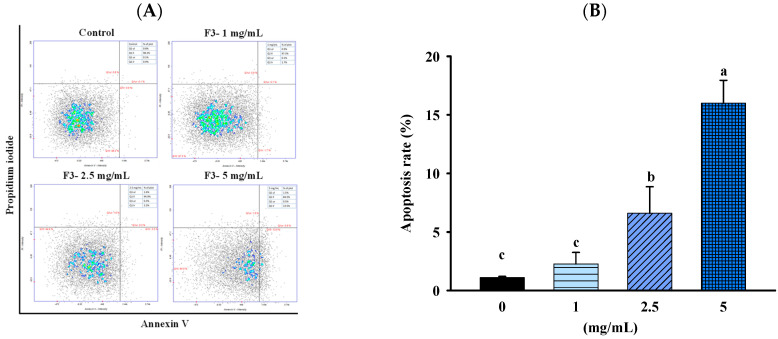
Effect of FBF on representative images of apoptosis analysis (**A**) and the levels of apoptosis (**B**) in HCT-116 cells. Data are expressed as the mean ± SD (*n* = 5). Different letter means that the values significantly differ from the others at *p* < 0.05 (Duncan’s multiple range tests). FBF: fish bone fermented with *Monascus purpureus* for 3 days.

**Figure 5:**
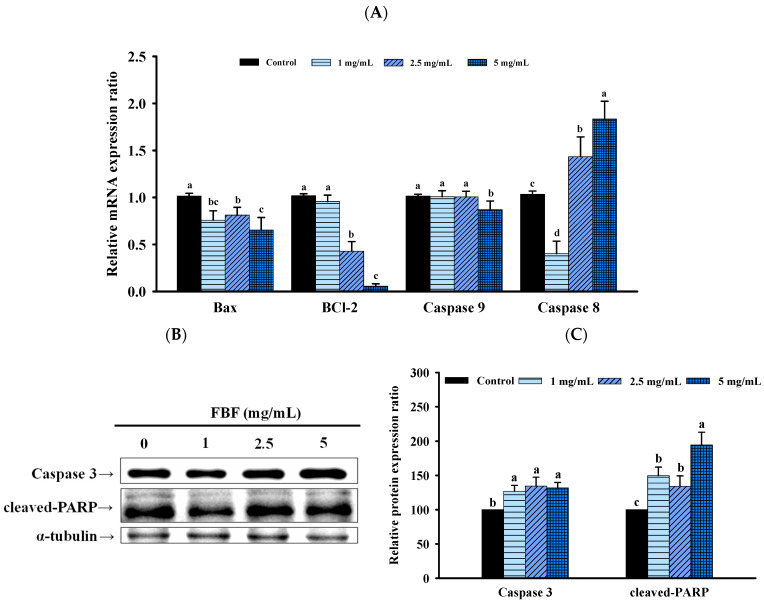
Effect of FBF on mRNA expression (**A**), representative Western blot images related to the apoptosis (**B**) and the Western blot quantification related to the apoptosis (**C**) in HCT-116 cells. Data are expressed as the mean ± SD (*n* = 5). Different letter means that the values significantly differ from the others at *p* < 0.05 (Duncan’s multiple range tests). FBF: fish bone fermented with *Monascus purpureus* for 3 days.

To confirm whether the inhibitory effect of FBF on HCT-116 cells was related to the induction of apoptosis, we quantified the apoptotic populations in the FBF-treated HCT116 cells stained with Annexin-V/propidium iodide using an NC-3000™ fluorescence image cytometer. As shown in Figure 4 and Figure 5, the apoptotic rate of HCT-116 cells increased significantly following FBF treatment. The percentages of apoptotic in the three groups of FBF-treated HCT-116 cells were 2.3%, 6.6% and 16.0% for 1, 2.5 or 5 mg/mL, respectively, while that of the control group was 4.0% (Figure 4). Furthermore, crucial functions in the apoptotic process are played by Bcl-2, Bax, caspases 3, 8 and 9 and PARP. Therefore, the expression levels of these apoptotic mRNA or proteins were determined using real-time PCR or Western blotting. The results show that 2.5 or 5 mg/mL of FBF have significantly upregulated the mRNA expression of caspases 8, while the Bcl-2, Bax and caspases 9 did not increase (Figure 5A). However, the protein expression of caspases 3 and cleaved-PARP were enhanced following different doses of FBF treatment (Figure 5B,C).

### 2.4. FBF Inhibits the Growth of HCT-116 Cells by Inducing Autophagy

**Figure 6:**
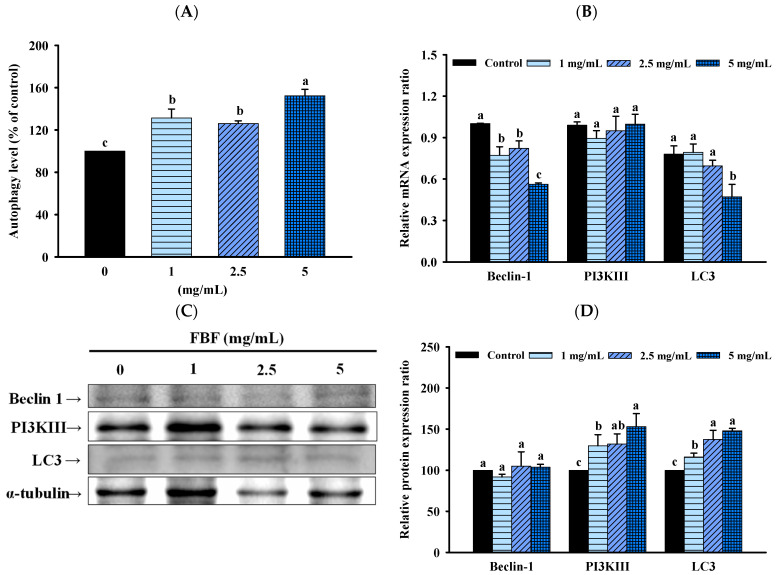
Effect of FBF on autophagy level (**A**), mRNA expression (**B**), representative Western blot images related to the autophagy (**C**) and the Western blot quantification related to the autophagy (**D**) in HCT-116 cells. Data are expressed as the mean ± SD (*n* = 5). Different letter means that the values significantly differ from the others at *p* < 0.05 (Duncan’s multiple range tests). FBF: fish bone fermented with *Monascus purpureus* for 3 days.

To confirm whether the inhibitory effect of FBF on HCT-116 cells was associated with the induction of autophagy, we quantified the autophagy populations in the FBF-treated HCT116 cells stained with Cyto-ID green stain using an NC-3000™ fluorescence image cytometer. As shown in Figure 6, the autophagy rate of HCT-116 cells increased significantly following different doses of FBF treatment. The percentages of autophagy in the three groups of FBF-treated HCT-116 cells were 131.4%, 126.2% and 152.4% for 1, 2.5 or 5 mg/mL, respectively (Figure 6A). Furthermore, Beclin 1, PI3K III and LC3 are crucial components of the autophagy process. Therefore, real-time PCR or Western blotting were used to determine the expression levels of these autophagy mRNA or proteins. The results show that the mRNA expression of Beclin 1, PI3K III and LC3 had no effect in HCT-116 cells following FBF treatment (Figure 6B). However, the different doses of FBF were enhanced following the protein expression of PI3K III and LC3, while Beclin 1 did not increase (Figure 6C,D).

## 3. Discussion

Studies have shown that a *Lactobacillus plantarum* KFY02 fermented grape skin extract solution (KFSE) [19] and grape skin fermentation using *Lactobacillus fermentum* CQPC04 (CF) [20] can inhibit the growth of the HepG2 cell line. MCF7, A549 and HCT-116 cell line growth can be inhibited using *Kluyveromyces marxianus* NRRL Y-8281 fermented pineapple waste (FPW) [21]. In this research, HCT-116 cell line growth can be inhibited using FBF. Furthermore, the effect was more pronounced by FBF when compared to the MP (*Monascus purpureus* that has an anti-cancer effect) in the same concentration. This study demonstrates the ability of fermented by-products to inhibit the growth of cancer cells.

The cell cycle is closely related to the formation of cancer. When the cell cycle is abnormally regulated and DNA is severely damaged, it will cause excessive cell proliferation and accumulation, eventually leading to cancer formation [22,23,24]. Studies on natural products or extracts in various cancer cells have shown that they inhibit the proliferation of cancer cells by inducing G2/M phase cell cycle arrest. The G2/M checkpoint is critical for preventing damaged DNA from entering mitosis, allowing time for repair or triggering apoptosis if the damage is irreparable [23,25]. For instance, aloperine and homogeneous polysaccharides induced cell cycle arrest in the G2/M phase of human colon cancer (HCT-116) cells [26,27]; quercetin induced G2/M arrest in human cervical cancer (HeLa) cells [28]; Licochalcone A induced G2/M arrest in human hepatoma (HepG2) cells [29]; and Germacrone induced G2/M arrest in human gastric cancer (BGC823) cells [30]. Additionally, previous research has demonstrated that fermentation can inhibit the growth of cancer cells by cell cycle arrest [31]. KFSE can induce cancer cell cycle arrest, thereby inhibiting HepG2 cell growth [19]. Building upon these findings, we investigated the impact of FBF on cell cycle progression in HCT-116 cells. This study is consistent with previous research, showing that FBF induces cell cycle arrest at the G2/M phase to inhibit the proliferation of HCT-116 cells. However, the cell cycle progresses through different stages: the S phase is the phase before cell division, during which DNA synthesis and histone synthesis occur before entering the G2/M phase [24,25,32]. Therefore, inhibiting DNA replication of cancer cells will halt the abnormal regulation of the cell cycle. This study demonstrates that FBF induces cell cycle arrest, specifically in the S phase, preventing the DNA replication of cancer cells and thereby inhibiting the proliferation and dissemination of cancer cells. The findings from these studies have important implications for cancer treatment. By inducing cell cycle arrest, natural products and fermentation may provide a promising approach to inhibiting cancer cell proliferation. However, the mechanisms underlying fermentation-induced cell cycle arrest and the potential limitations of this approach warrant further investigation.

Apoptosis is a natural mechanism of cell death and one of the targets of anti-cancer therapy [33]. Apoptotic pathways are mainly divided into intrinsic (mitochondrial stress-mediated) and extrinsic (death receptor-mediated) pathways, both of which use caspase as the intersection. In caspases, caspase 3 is a key executioner in the apoptotic pathway, and it can be activated by the promoters of caspases, such as caspases 8 and 9. On the apoptotic pathway, caspase 8 plays a significant role in the death receptor-mediated pathway, whereas caspase 9 plays an important role in the mitochondrial pathway. Once activated, caspase 8 activates the downstream key factor caspase 3 and subsequently initiates apoptosis [34,35]. Studies have shown that KFSE can induce cancer cell apoptosis by regulating the protein expression of Bax and caspase 8, thereby inhibiting HepG2 cell growth [19]. CF can induce cancer cell apoptosis by regulating the mRNA expression of Bax and protein expression of caspase 3, 7, 8 and 9, thereby inhibiting HepG2 cell growth [20]. Fermented wheat germ can induce cancer cell apoptosis by regulating caspase 3, 7 and 9 activity and the protein expression of PARP, thereby inhibiting SK-OV-3 and OVCAR-3 cell growth [36]. *Lactobacillus plantarum* DGK-17-fermented soybean seed extract can induce cancer cell apoptosis by regulating the protein expression of caspase 9, 3, BCl-2 and Bax, thereby inhibiting HCT-116 cell growth [37]. Previous studies show that fermentation induces apoptosis of cancer cells, mainly through the regulation of the expression of caspase 3, 7, 8, 9, BCl-2 and Bax. In the present study, we found that FBF effectively activated caspase 8, caspase 3 and PARP in HCT-116 cells, while Bax and caspase 9 expression was not affected. It indicated that FBF-induced apoptosis of HCT-116 cells was closely related to the death receptor-mediated pathway. The activation of caspase 8 by FBF suggests a potential therapeutic strategy for cancers sensitive to death receptor-mediated apoptosis. The novelty of our study lies in uncovering the specific apoptotic pathway regulated by FBF, highlighting its unique mechanism of action. Therefore, our study adds to the growing body of evidence supporting the potential of natural compounds as effective anti-cancer therapies through apoptotic pathways, contributing to the broader understanding of apoptosis regulation.

Fermentation can encourage cancer cell autophagy, which inhibits the growth of cancer cells. Fermented wheat germ extract can induce cancer cell autophagy by regulating the protein expression of LC3, thereby inhibiting HRT-18 cell growth [38]. Our results are similar to those of previous studies: FBF induces autophagy in HCT-116 cells by regulating the protein expression of PI3KIII and LC3. These findings are consistent with previous research demonstrating that fermentation can induce autophagy in cancer cells, providing another mechanism through which fermented products may inhibit cancer growth.

The concentration of FBF used in this study is higher than that of other fermented products (such as fermented wheat germ (2000 μg/mL), fermented blueberries (100 μM) and fermented curly kale (50 μg/mL)) extracted for anti-cancer research. This may be due to the fact that these fermented products have been extracted after fermentation to increase the active ingredients, while the FBF used in this study was unextracted [36,39,40]. In addition, it was observed that some substances with anti-cancer activity (such as geraniol, vanillin and sesamol) were used at concentrations ranging from 2 to 5 mM [41,42,43]. This study (2.5–5 mM of FBF) was similar to previous studies; therefore, we believe that FBF has anti-cancer potential.

Studies have demonstrated that histidine, glutamic acid, aspartic acid and proline have anti-cancer effects [44]. Under acidic conditions, histidine can cause cytotoxicity in cancer cells by increasing membrane permeability; aspartic and glutamic acid residues may have an inhibitory effect on the proliferation of tumor cells; proline interacts with membranes and has structural flexibility, both of which may help it acquire more cytotoxic effects [44]. The contents of histidine, glutamic acid, aspartic acid and proline in fish bone are 10.10 ± 0.3, 1.80 ± 0.1, 0.06 ± 0.0 and 0.55 ± 0.1 mg/100 mL, respectively. After fermentation, histidine content 25.76 ± 1.5, glutamic acid content 5.73 ± 0.3, aspartic acid content 1.16 ± 0.1 and proline content 1.19 ± 0.1 mg/100 mL were present in FBF [17]. The histidine, glutamic acid, aspartic acid and proline content in the fish bone can be increased, respectively, by 155, 218, 1833 and 116% through fermentation. According to these findings, the anti-cancer properties of fermented products may be attributed to increasing the amount of these anti-cancer amino acids after fermentation.

In this study, we investigated the potential anti-cancer properties of FBF, a compound that has not been previously studied in this context. Due to the unique nature of FBF as an unexplored candidate, we chose not to include a positive control group. While this decision limits direct comparisons with established anti-cancer agents, it allowed us to focus on understanding FBF’s intrinsic effects independently. By doing so, we aimed to highlight FBF’s distinct potential as a novel anti-cancer agent. The absence of a positive control group should be acknowledged as a limitation in our study. Future research incorporating positive controls will be valuable to provide a more comprehensive evaluation of FBF’s relative efficacy compared to clinically applied anti-cancer agents. Despite this limitation, our findings offer valuable insights into the unexplored potential of FBF as an anti-cancer compound and pave the way for further investigations in this promising area of research.

## 4. Materials and Methods

### 4.1. Chemicals and Reagents

Phenylmethanesulfonyl fluoride (PMSF), McCoy’s 5A medium, Dulbecco’s Modified Eagle Medium (DMEM), tris-base, sodium chloride, bovine serum albumin (BSA), TRIzol^®^ reagent, tween 20, 3-(4,5-dimethylthiazol-2-yl)-2,5-diphenyl tetrazolium bromide (MTT), and sodium azide were purchased from Sigma-Aldrich (St. Louis, MO, USA). Phosphate-buffered saline (PBS) was purchased from Unionward (Taipei, Taiwan). Trypsin-EDTA, penicillin/streptomycin and fetal bovine serum (FBS) were purchased from Gibco (Grand Island, NY, USA). Recombinant RNasin ribonuclease inhibitor, oligo d(T)21 and M-MLV reverse transcriptase were purchased from Promega (Madison Avenue, New York City, NY, USA). 4′,6-diamidino-2-phenylindole (DAPI), lysis buffer, stabilization buffer and Hoechst 33,342 were purchased from ChemoMetec A/S (Gydevang, Allerod, Denmark). CYTO-ID^®^ autophagy detection kit was purchased from Enzo Life Sciences (cat. no. ENZ-51031; Madison Avenue, NY, USA). Chemiluminescence (ECL) kit was purchased from Bio-Rad (cat. no. 1705061, Hercules, CA, USA).

### 4.2. Preparation of FBF

The *Monascus purpureus* BCRC 31,499 was purchased from the Bioresources Collection and Research Center (BCRC; Hsinchu, Taiwan). The bone of a milkfish (*Chanos chanos*) was purchased from an aquatic supplier (Kaohsiung, Taiwan). Fish bone fermented with *M. purpureus* for 3 days (FBF) was prepared, based on the approach of our previous study [17].

### 4.3. Cell Culture and Treatment

The human colorectal cancer cell line (HCT-116 cells) and the rat kidney epithelial cell line (NRK-52E cells) were obtained from the BCRC (Hsinchu, Taiwan). HCT-116 cells were grown in McCoy’s 5A medium (containing 10% FBS and 1% penicillin/streptomycin) at 37 °C for 5% CO_2_. The cytotoxicity assay was divided into six groups: control (untreated), FB (5 mg/mL), *Monascus purpureus* (MP; 5 mg/mL) and FBF (1, 2.5, or 5 mg/mL), respectively. The cells were cultured in a 3.5 cm dish at a density of 5 × 10^5^ cells/mL and treated with control, FB, MP and different concentrations of FBF for 24 h. For cell viability, they were divided into control (untreated) and 1, 2.5 or 5 mg/mL of FBF. The NRK-52E cells were cultured in a 3.5 cm dish at a density of 5 × 10^5^ cells/mL and treated with control and different concentrations of FBF for 24 h. For cell proliferation, apoptosis and autophagy assays, they were divided into four groups: control and FBF (1, 2.5 or 5 mg/mL), respectively. The cells were cultured in a 3.5 cm dish at a density of 5 × 10^5^ cells/mL and treated with control and different concentrations of FBF for 24 h.

### 4.4. Cell Viability Analysis

Cell viability was determined following the MTT method described by Denizot and Lang [45]. The MTT assay was performed using the method that was previously described [17].

### 4.5. Cell Cycle Distribution Analysis

The NucleoCounter^®^ NC-3000^TM^ fluorescence imaging cytometer was utilized to detect the distribution of the cell cycle (Chemometec, Allerod, Denmark) following incubation with control, 1, 2.5 or 5 mg/mL of FBF for 24 h. The cells were washed with PBS twice, then recovered by detaching cells with 100 μL of 0.25% trypsin-EDTA and rinsing in a medium. The supernatant was discarded after the cell suspension was centrifuged (860× *g* for 5 min, 25 °C). The supernatant was discarded after the cells had been centrifuged (860× *g* for 5 min, 25 °C) and cleaned with PBS once. A quantity of 8 × 10^5^ cells/mL of cells was lysed in 100 μL of lysis buffer with 2 μL of 10 μg/mL DAPI at 37 °C for 5 min. Subsequently, 100 μL of stabilization buffer was added. Slide-A8 was infused with 10 μL of the cell solution for NucleoCounter^®^ NC-3000^TM^ (Chemometec, Allerod, Denmark) fluorescence imaging cytometer detection.

### 4.6. Apoptosis Percentage Analysis

Following the incubation with control, 1, 2.5 or 5 mg/mL of FBF for 24 h, the NucleoCounter^®^ NC-3000™ fluorescence image cytometer was used for the detection of apoptotic percentages of cells stained with annexin-V FITC. The cells were washed with PBS twice, then recovered by detaching cells with 100 μL of 0.25% trypsin–EDTA and rinsing in a medium. The supernatant was discarded after the cell suspension was centrifuged (201× *g*, 25 °C for 5 min). The cells were incubated at 25 °C for 15 min in the dark after being suspended in 100 μL of binding buffer (5 μL of annexin-V FITC and 1 μL of PI). Then, the sample was centrifuged at 201× *g* for 5 min, 25°C; the supernatant was removed, and the sample was dissolved in 100 μL of PBS before 2 μL of 500 μg/mL Hoechst 33,342 was added and reacted for 5 min at 37 °C. Then, the sample was centrifuged (201× *g*, 25 °C, 5 min), the supernatant was discarded, the cells were washed three times with 300 μL of PBS and the pellet was dissolved in 100 μL of PBS. A quantity of 30 μL of the cell solution was infused to Slide-A2 for detection using NucleoCounter^®^ NC-3000™ fluorescence image cytometer.

### 4.7. Autophagy Percentage Analysis

Measurement of the autophagy rate in cells was determined using the NucleoCounter^®^ NC-3000^TM^ fluorescence imaging cytometer and a CYTO-ID^®^ autophagy detection kit following incubation with control and 1, 2.5 or 5 mg/mL of FBF for 24 h. The cells were washed with PBS twice, then recovered by detaching cells with 100 μL of 0.25% trypsin-EDTA and rinsing in a medium. The supernatant was discarded after the cell suspension was centrifuged (860× *g* for 5 min, 25 °C) and cleaned with PBS once. The cell count of 1 × 10^6^ cells/mL of cells was dissolved in 200 μL of PBS; subsequently, 0.4 μL of Cyto-ID green stain solution was added at 37 °C for 5 min in the dark. The sample was centrifuged at 860× *g* for 5 min at 25 °C; the supernatant was removed before the cells were dissolved in 100 μL of PBS. A quantity of 30 μL of the cell solution was infused to Slide-A2 for detection using NucleoCounter^®^ NC-3000™ fluorescence image cytometer.

### 4.8. Relative mRNA Expression Analysis

Real-time PCR was used to detect the mRNA expression in the HCT-116 cells. Briefly, the cells were cultured in a 3.5 cm dish at a density of 5 × 10^5^ cells/mL overnight and then treated with control and 1, 2.5 or 5 mg/mL of FBF for 24 h. Using the TRIzol^®^ reagent and following the manufacturer’s instructions, total RNA was extracted from the cells. According to the manufacturer’s instructions, oligo d(T)21 was used as a primer with recombinant RNasin ribonuclease inhibitor, and, along with M-MLV reverse transcriptase, were employed to synthesize the first strand of complementary cDNA. Then, a real-time PCR assay was carried out according to the previously described method^18^. The normalized gene-specific primer was β-actin, and the target gene primers are shown in Table 1. The data were processed using the LightCycler^®^ 96 Application Software Version 1.1 (Roche Diagnostics, Penzberg, Germany), and the relative expression of the target genes was computed using the 2^−ΔΔCt^ method.

### 4.9. Western Blot Analysis

The relative protein expression level of poly (ADP-ribose) polymerase (PARP), beclin 1, caspase 3, microtubule-associated protein 1B light chain 3 (LC3) and phosphoinositide 3 kinase III (PI3KIII) were determined using a Western blot analysis. Briefly, the cells were cultured in a 3.5 cm dish at a density of 5 × 10^5^ cells/mL overnight and then treated with control and 1, 2.5 or 5 mg/mL of FBF for 24 h. The total protein was extracted from cells with a mixture of lysis buffer (containing 12 mM dipotassium hydrogen phosphate, 0.5 mM PMSF and 8 mM potassium dihydrogen phosphate, pH 7.4). The total protein concentration was determined following the method described by Lowry et al. [46]. The Western blot study employed a total of 20 μg of cellular protein lysate per lane. Proteins were separated on a 15% sodium dodecyl sulphate polyacrylamide gel. The proteins were then moved to polyvinylidene difluoride (PVDF) membranes following electrophoresis. Non-specific protein interactions were blocked by the blocking buffer (containing 125 mM sodium chloride, 1% BSA, 0.2% tween 20, 20 mM tris-base, 0.1% sodium azide, pH 7.4) for 1 h at room temperature. The membranes were then incubated with an Anti-PARP (1:200 dilution, cat. no. 436400, Invitrogen, Carlsbad, CA, USA), Anti-Caspase 3 (1:1000 dilution, cat. no. 9662, Cell Signaling Technology, Danvers, MA, USA), Anti-Beclin 1 (1:1000 dilution, cat. no. 3495, Cell Signaling Technology, Danvers, MA, USA), anti-LC3 (1:1000 dilution, cat. no. ABC232, Millipore, Milford, CT, USA), Anti-PI3KIII (1:1000 dilution, cat. no. 3811, Cell Signaling Technology, Danvers, MA, USA), Anti-GAPDH (1:10,000 dilution, cat. no. GTX100118, GeneTex, Hsinchu City, Taiwan) and Anti-α-tubulin (1:500 dilution, cat. no. 405306, BioLegend, San Diego, CA, USA) at 4 °C overnight. On the second day, the PVDF membranes were incubated in a solution of anti-mouse IgG (1:5000, 405306, BioLegend, San Diego, CA, USA) or goat anti-rabbit IgG (1:10,000, 111-035-003, Jackson ImmunoResearch, PA, USA) for 1 h at room temperature. α-tubulin or GAPDH was used as an endogenous control. Following the manufacturer’s instructions, protein bands were detected using an ECL kit, and their quantities were determined using a ChemiDocTM MP Imaging System (Bio-Rad Laboratories Inc., Hercules, CA, USA). Data were analyzed with ImageLab_5.1 software (Bio-Rad Laboratories Inc.).

### 4.10. Statistical Analysis

The data are shown as the means ± standard deviation. The program SPSS software (SPSS version 12.0, Inc., Chicago, IL, USA) was used for the statistical analysis. The data were examined using a one-way analysis of variance (ANOVA) followed by Duncan’s multiple range tests or Tukey’s tests. A *p* < 0.05 was considered statistically significant.

## 5. Conclusions

In conclusion, the present investigation showed that FBF inhibits the growth of HCT-116 cells. The underlying anti-colorectal cancer mechanism of FBF might be inducing the death receptor-mediated apoptotic pathway, the autophagy pathway and cell cycle arrest. Therefore, this study indicates that FBF has potential in the prevention and treatment of colorectal cancer. Nevertheless, further research and validation are needed to ascertain the safety and efficacy of FBF in clinical applications. Future work should delve deeper into FBF’s mechanisms and compare them with other treatment modalities to enhance our understanding of colon cancer treatment. In summary, we look forward to further research that will translate these findings into practical applications, ultimately benefiting colon cancer patients.

## 6. Patents

This research obtained Taiwan invention patent, patent cert. No.: I706728.

## Figures and Tables

**Table 1:** Specific primer of real-time PCR on human genes in this study.

Gene	Primer sequence (5′-3′)	Reference (NCBI GenBank)
β-actin	F-ATGTGCAAGGCCGGCTTC R-GAATCCTTCTGACCCATGCC	NM_001101.3
Bax	F-TGTTTTCTGACGGCAACTTCA R-AGCCCATGATGGTTCTGATCA	NM_001291428.1
Bcl-2	F-CCTGTGGATGACTGAGTACCTGAAC R-CAGCCAGGAGAAATCAAACAGA	NM_000633.2
Caspase 8	F-TCCAAATGCAAACTGGATGATG R-TTTTCAGGATGTCCAACTTTCCTT	NM_001080124.1
Caspase 9	F-AGCTGGACGCCATATCTAGTTTG R-AACGTACCAGGAGCCACTCTTG	NM_001229.4
PI3K III	F-TTGGAGACAGGCACCTGGAT R-CCATTTCTTTATTCAGCTTCATTGG	NM_001308020.1
Beclin 1	F-CTGGACACGAGTTTCAAGATCCT R-GTTAGTCTCTTCCTCCTGGGTCTCT	NM_001313998.1
LC3	F-TCCTGGACAAGACCAAGTTTTTG R-ACCATGCTGTGCTGGTTCAC	NM_032514.3

## Data Availability

Not applicable.

## Supplementary Materials

The following supporting information can be downloaded at https://www.mdpi.com/article/10.3390/molecules28155679/s1, Figure S1: Effect of F3 on representative images of cell cycle analysis in HCT-116 cells.

## Sample Availability

Samples of the compounds are not available from the authors.
