# Supplementary material for: Exploring the Anti-Cancer Effects of Fish Bone Fermented Using Monascus purpureus: Induction of Apoptosis and Autophagy in Human Colorectal Cancer Cells
Source: Molecules. 2023 Jul 27;28(15):5679. doi: 10.3390/molecules28155679 (PMC10419882; doi:10.3390/molecules28155679)
Supplement: Supplementary file 1 [file molecules-28-05679-s001.zip › molecules-2501892-supplementary.pdf]

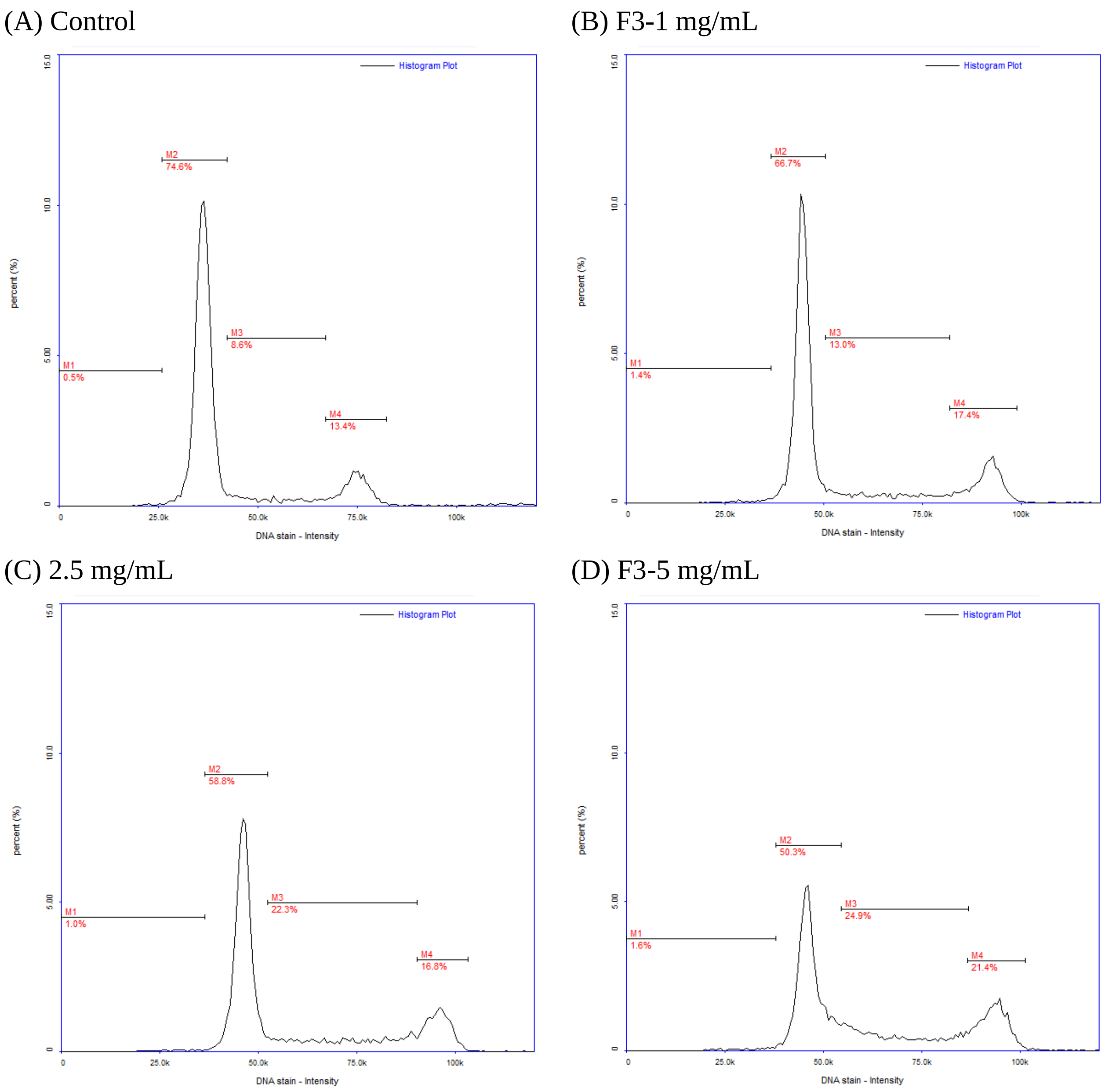

Supplementary Figure S1. Effect of F3 on representative images of cell cycle analysis in HCT-116 cells. Data are expressed the mean ± SD (n = 5). FB: Fish bone; MP: *Monascus purpureus*; FBF: fish bone fermented with *Monascus purpureus* for 3 days.
